# Genetically Low Antioxidant Protection and Risk of Cardiovascular Disease and Heart Failure in Diabetic Subjects

**DOI:** 10.1016/j.ebiom.2015.11.026

**Published:** 2015-11-14

**Authors:** Camilla J. Kobylecki, Shoaib Afzal, Børge G. Nordestgaard

**Affiliations:** a Department of Clinical Biochemistry, Herlev and Gentofte Hospital, Copenhagen University Hospital, Herlev Ringvej 75, DK-2730 Herlev, Denmark; b Department of Clinical Biochemistry, Rigshospitalet, Copenhagen University Hospital, Blegdamsvej 9, 2100 Copenhagen, Denmark

**Keywords:** Superoxide dismutase 3, Cardiovascular disease, Diabetes, Oxidative stress

## Abstract

**Background:**

Hyperglycemia-induced oxidative stress is one mechanism believed to underlie diabetic vascular disease. We tested the hypothesis that diabetic subjects heterozygous for extracellular superoxide dismutase (SOD3) R213G, which entails lower antioxidant capacity in tissues, have increased risk of cardiovascular disease and heart failure.

**Methods:**

We used the prospective Copenhagen General Population Study and Copenhagen City Heart Study and genotyped 95,871 individuals for the rs1799895 R213G variation in the *SOD3* gene, of which 4498 had diabetes. We used national hospitalization and death registers to assess cardiovascular disease and heart failure.

**Findings:**

Out of 95,871 individuals, we identified 93,521 R213G non-carriers (213RR, 97.5%), 2336 heterozygotes (213RG, 2.4%) and 14 homozygotes (213GG, 0.01%). In diabetic subjects, the hazard ratio for cardiovascular disease in R213G heterozygotes compared to non-carriers was 2.32 (95% CI 1·44–3.75), with a corresponding hazard ratio in non-diabetic subjects of 0.97 (0·80–1.19) (p for interaction 0.002). For heart failure, the hazard ratios in R213G heterozygotes compared to non-carriers were 2.19 (1.28–3.76) in diabetic and 0.68 (0.49–0.92) in non-diabetic subjects (p for interaction < 0.001).

**Interpretation:**

Risk of cardiovascular disease and heart failure was higher in R213G heterozygotes versus non-carriers in diabetic subjects, but not in non-diabetic subjects.

## Highlights

• SOD3 R213G heterozygosity was associated with a 2-fold higher risk of cardiovascular disease and heart failure.
• This association was observed in diabetic subjects, but not in non-diabetic subjects.
• This could provide further insight on the pathophysiology of cardiovascular disease and heart failure in diabetes.

Diabetic patients have high risk of cardiovascular disease, possibly due to increased oxidative stress. The SOD3 enzyme is an important extracellular antioxidant in blood vessel walls. A rare mutation in the *SOD3* gene causes the enzyme to diffuse from vessel walls into the blood stream. In diabetic subjects, we found a 2-fold higher risk of cardiovascular disease and heart failure among those with the rare SOD3 variant compared to those with the normal variant, with no effect of the variant among non-diabetic subjects. This could help provide further insight on the mechanisms underlying cardiovascular disease and heart failure in diabetes.

## 1 Introduction

Cardiovascular disease is the leading cause of morbidity and mortality in individuals with diabetes, with a 2–4 fold higher risk of cardiovascular disease and up to 3 fold higher risk of mortality compared to those without diabetes (Fox, CS, et al., 2004, Preis, SR, et al., 2009). Hyperglycemia-induced oxidative stress with subsequent increase in oxidized biomolecules and associated tissue damage is one mechanism believed to underlie diabetic vascular disease (Schaffer, SW, et al., 2012, Fiorentino, TV, et al., 2013). Furthermore, oxidative stress has been implicated in the atherosclerotic process as well as in myocardial damage (Maiolino, G, et al., 2013, Waddingham, MT, et al., 2015). As a result, myocardial damage may lead to myocardial dysfunction and heart failure, a common diabetic complication that can develop independently of other cardiovascular risk factors (Waddingham et al., 2015).

The extracellular superoxide dismutase enzyme (SOD3, EC-SOD) is the major extracellular scavenger of superoxide anions and regulates vascular superoxide anion levels by catalyzing the dismutation of superoxide into oxygen and hydrogen peroxide (McCord and Fridovich, 1969). SOD3 is mainly secreted by vascular smooth muscle cells and shows high affinity for heparan sulfate proteoglycans, other sulfated proteoglycans as well as type I collagen, and thus, is largely bound to cell surfaces and extracellular matrix components in blood vessel walls (Stralin, P, et al., 1995, Oury, TD, et al., 1996, Petersen, SV, et al., 2005). The tissue-bound form is believed to make up 90–99% of total SOD3 (Sandström et al., 1993). The SOD3 binding to heparin–heparan sulfate molecules is electrostatic in nature and mediated through an extracellular matrix-binding domain, where a cluster of 6 positively charged amino acids in position 210–215 in the C-terminal end forms the essential part (Sandstrom et al., 1992). A rare mutation in the extracellular-binding domain of SOD3 at position 213 (arginine to glycine, R213G) leads to decreased extracellular matrix affinity with unaltered enzymatic activity (Sandstrom et al., 1994). The decreased extracellular matrix affinity in R213G is likely caused by interruption of the ionic interaction with heparin–heparan sulfate as well as by altered tertiary structure of the enzyme, disrupting the collagen binding capacity (Petersen et al., 2005). Consequently, R213G heterozygotes have up to 10-fold higher plasma levels of SOD3 and blood vessel walls that are deficient in the SOD3 enzyme (Sandstrom et al., 1994).

We hypothesized that the antioxidant SOD3 enzyme in diabetic subjects plays an especially important role in protecting the vessel walls from oxidative damage. Thus, in the present study we tested the hypothesis that diabetic subjects heterozygous for the SOD3 R213G genetic variation, which entails lower antioxidant capacity in tissues, have increased risk of cardiovascular disease and heart failure. For this purpose we genotyped 95,871 individuals from the Danish general population, of which 4498 had diabetes.

## 2 Methods

### 2.1 Study Population

We used the Copenhagen General Population Study (CGPS), initiated in 2003 with ongoing enrollment, and the Copenhagen City Heart Study (CCHS), initiated in 1976–1978 with follow-up examinations in 1981–1983, 1991–1994, and 2001–2003 (Nordestgaard, BG, et al., 2007, Thomsen, M, et al., 2013). DNA was available in the CGPS and in the 1991–1994 and 2001–2003 examinations of the CCHS. For both studies, individuals aged 20–100 years were invited randomly from the Danish Civil Registration System to reflect the Danish general population. Participation rate was 43% in the CGPS and 61% and 50% in the CCHS 1991–1994 and 2001–2003 examinations. The study was approved by Herlev and Gentofte Hospital and by Danish Ethical Committees, and was conducted according to the Declaration of Helsinki. Written, informed consent was obtained from all participants. No subject was lost to follow-up. We only included white individuals of Danish descent with DNA available, a total of 87,030 individuals from the CGPS and 8841 from the CCHS.

### 2.2 Cardiovascular Disease and Heart Failure

Information on diagnosis of myocardial infarction (ICD8: 410, ICD10: I21–I22), heart failure (ICD8: 427.09–427.11; ICD10: I50.0–I50.9) and ischemic stroke (ICD8: 433–434, ICD10: I63) were collected from 1977 through April 2013 from the national Danish Patient Registry and the national Danish Causes of Death Registry, while the date of death was obtained from the Danish Civil Registration System, as done previously (Nordestgaard, BG, et al., 2007, Kamstrup, PR, et al., 2009, Nordestgaard, BG, et al., 2012). The national Danish Causes of Death Registry ranks main causes of death as well as contributing causes of death, as reported by the attending physician, or physicians in a forensic or pathology department. Cardiovascular disease was a composite of the endpoints myocardial infarction, ischemic stroke and death from cardiovascular disease, whichever endpoint came first; a death was classified as due to cardiovascular disease if one of three ranked causes of death had a cardiovascular diagnosis (ICD8: 390–458, ICD10: I00–I99), as done previously (Afzal et al., 2014).

### 2.3 Diabetes

Baseline diabetes was defined as self-reported diabetes of any type, a hospital diagnosis of diabetes prior to the examination (ICD8: 249–250, ICD10: E10, E11, E13, E14), non-fasting plasma glucose > 11 mmol/L at examination, or use of antidiabetic medication. Baseline and hospitalization diabetes included both individuals with baseline diabetes and individuals who received a register diabetes diagnosis of any type during follow-up. Baseline diabetes included 4498 individuals, of which 464 experienced a cardiovascular event prior to examination, leaving 4034 individuals for prospective analyses. For heart failure, 208 diabetic subjects received a heart failure diagnosis prior to examination, leaving 4290 for prospective analyses. When individuals who did not have diabetes at the time of examination, but who received a diabetes diagnosis during follow-up, were included, a total of 5145 individuals with diabetes were eligible for prospective analyses.

### 2.4 Covariates

Baseline characteristics were recorded from a self-administered questionnaire, a physical examination, and from blood samples. Participants reported on smoking status (never, former, current) and if relevant, number of years of smoking and daily tobacco consumption, from which cumulative tobacco consumption in pack-years was calculated; a pack-year was 20 cigarettes or equivalent smoked per day for one year. Non-smokers were defined as never or previous smokers. Self-reported weekly alcohol intake was in units of ~ 12 g of alcohol. Furthermore, information on weekly physical activity (highest versus lowest half of physical activity groups) and level of income (highest third versus lowest two thirds), were obtained from the questionnaire. Body mass index (BMI) was calculated from measured weight in kilograms divided by measured height in meters squared. Systolic blood pressure was measured. We defined hypertension as systolic blood pressure ≥ 140 mm Hg (diabetics ≥ 130 mm Hg), diastolic blood pressure ≥ 90 mm Hg (diabetics ≥ 80 mm Hg), or use of antihypertensive medication at time of examination. The data was > 99% complete and missing values for covariates were imputed according to age and sex to obtain a complete dataset; however, if individuals with any missing data were excluded, results were similar to those presented.

### 2.5 Laboratory Analyses

In the CGPS, we genotyped 87,030 individuals for the *SOD3* rs1799895 (R213G) variant, using a TaqMan-based assay (Applied Biosystems) and TaqMan GenoTyper v1.2 with a call rate of 99.3%. We used DNA extracted from leukocytes in peripheral blood using the Qiagen blood kit for DNA extraction. Genotypes were assigned in smaller batches, each containing around 5500 individuals. In the CGPS, a total of 87,718 individuals were attempted genotyped, 87,030 successfully. In the CCHS, 8841 individuals out of 9251 had been genotyped successfully earlier, using polymerase chain reaction followed by restriction enzyme digestion and agarose gel electrophoresis (RFLP), and all R213G carriers (213RG and 213GG) had been reanalyzed and confirmed by DNA sequencing (Juul et al., 2004). We used R213G heterozygotes and homozygotes previously identified by RFLP and DNA sequencing as controls for the TaqMan based assay.

Plasma concentrations of triglycerides, low-density lipoprotein cholesterol, high-density lipoprotein cholesterol and high sensitive C-reactive protein were measured with standard hospital assays (Stender et al., 2013). Plasma SOD3 were measured using ELISA, and results have been reported previously (Juul et al., 2004).

### 2.6 Statistical Analyses

We used Stata v.13.1. A chi-square test evaluated Hardy–Weinberg equilibrium. Kruskal–Wallis test was used when comparing two samples. In all analyses, we combined the two studies in order to maximize statistical power, and we adjusted for study. We pooled type 1 and type 2 diabetes because we did not have information on type of diabetes from baseline questionnaire, and in the analyses that included hospital diagnoses of diabetes, we upheld the pooling in order to maximize statistical power.

To examine the association between SOD3 R213G heterozygosity and cardiovascular disease and heart failure, we used Cox proportional hazards regression models with entry at examination date and age as underlying time scale (referred to as age-adjusted), to estimate hazard ratios with 95% confidence intervals. Since all measured confounders were evenly distributed among genotypes, we adjusted only for age. Interaction was tested for using a likelihood ratio test by introducing a two-factor interaction term in a model also including both factors, e.g. diabetes and R213G genotype. Follow-up ended April 2013 and those dying or emigrating (n = 292) during follow-up were censored at their death or emigration dates, respectively. Test for proportionality of hazards over time was performed using graphical methods and residuals; no major violations were observed.

We calculated cumulative incidences of cardiovascular disease using competing-risks survival regression with the method of Fine and Gray, and calculated a subhazard ratio for cardiovascular disease, accounting for the competing risk of death. We used the STATA command stsplit to split records at either date of examination (for baseline diabetes) or at time of diagnosis of diabetes during follow-up, thus creating two episodes for each subject that developed diabetes during follow-up, in order to assess a possible different effect of the R213G variant on cardiovascular disease before and after occurrence of diabetes.

## 3 Results

Among the 95,857 individuals from the CCHS and CGPS combined, 4498 (5%) had diabetes at baseline (Table 1). During follow-up, 4581 individuals (5%) experienced a cardiovascular event and 2736 (3%) received a diagnosis of heart failure; for diabetic subjects, the corresponding frequencies were 11% (427/4034) and 8% (332/4290). 1098 individuals experienced both cardiovascular disease and heart failure during follow-up. The frequency of diabetes did not differ between rs1799895 (R213G) heterozygotes and non-carriers, nor did any baseline characteristics (Table 1); however, as previously shown plasma extracellular superoxide dismutase was 9-fold higher in heterozygotes compared to non-carriers in the CCHS (Juul et al., 2004). R213G genotype frequencies were 97.5% (93,521/95,871) for non-carriers (213RR) and 2.4% (2336/95,871) for R213G heterozygotes (213RG); R213G homozygotes (213GG, 0.01%, 14/95,871) were excluded from the analyses because of insufficient statistical power for homozygotes alone. The genotype distribution was in Hardy–Weinberg equilibrium (p = 0.88). In the two studies combined, mean follow-up time was 6 years (min 0.003 years, max 21.5 years).

### 3.1 Cardiovascular Disease

**Fig. 1:**
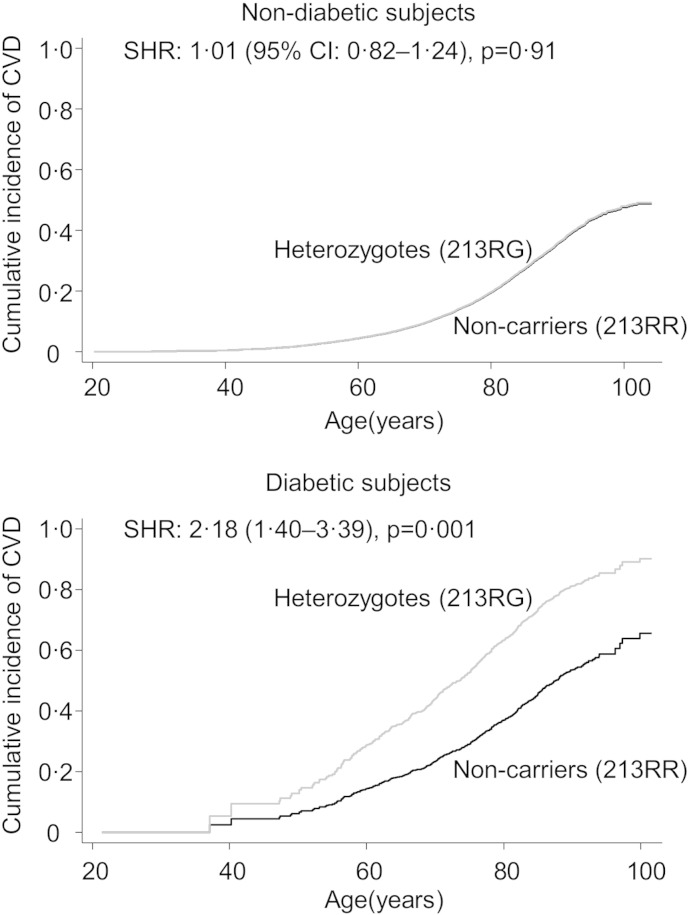
Cumulative incidence of cardiovascular disease by R213G SOD3 genotype, stratified by diabetes status. Diabetes included baseline diabetes and diabetes during follow-up from hospital registers. Baseline diabetes: self-reported diabetes, hospital diagnosis of diabetes prior to examination, non-fasting plasma glucose > 11 mmol/L at examination, or use of antidiabetic medication. CVD: cardiovascular disease, composite endpoint of cardiovascular death, myocardial infarction and ischemic stroke. Cumulative incidences and subhazard ratios (SHR) are from competing risk regression (Fine and gray). Stsplit at date of diabetes was used. For baseline diabetes, stsplit was at examination date. All analyses were adjusted for age and study.

**Fig. 2:**
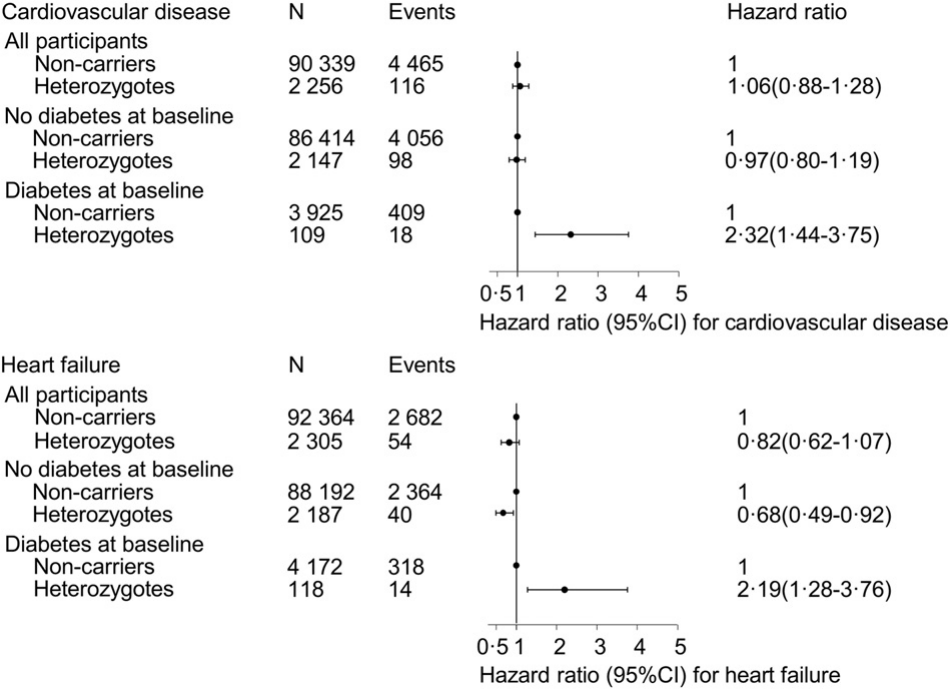
Risk of cardiovascular disease and heart failure by SOD3 R213G genotype, stratified by diabetes status. Non-carriers: 213RR. Heterozygotes: 213RG. Baseline diabetes: self-reported diabetes, hospital diagnosis of diabetes prior to examination, non-fasting plasma glucose > 11 mmol/L at examination, or use of antidiabetic medication. A total of 3262 individuals had experienced a cardiovascular event and 1188 had experienced heart failure prior to examination and were not included in the Cox regression with entry at examination. All estimates were adjusted for age and study. Cardiovascular disease was the composite endpoint of cardiovascular death, myocardial infarction, and ischemic stroke. CI: confidence interval.

The cumulative incidence of cardiovascular disease in R213G heterozygotes was higher than in non-carriers in diabetic subjects (sub-hazard ratio: 2.18, 95% CI: 1.40–3.39, p = 0.001), but not in non-diabetic subjects (SHR: 1.01 (0.82–1.24), p = 0.91) (Fig. 1). In Cox regression models, the hazard ratios for cardiovascular disease in R213G heterozygotes versus non-carriers were 2.32 (1.44–3.75) in diabetic subjects and 0.97 (0.80–1.19) in non-diabetic subjects (p for interaction = 0.002) (Fig. 2). These hazard ratios were similar in the CCHS and CGPS separately (Supplemental Fig. 1).

### 3.2 Heart Failure

The hazard ratios for heart failure in R213G heterozygotes versus non-carriers were 2.19 (95% CI: 1.28–3.76) in diabetic subjects and 0.68 (0.49–0.92) in non-diabetic subjects (p for interaction < 0.001) (Fig. 2). These hazard ratios were similar when analyzing the CCHS and CGPS separately (Supplemental Fig. 1).

### 3.3 Sensitivity Analyses

**Fig. 3:**
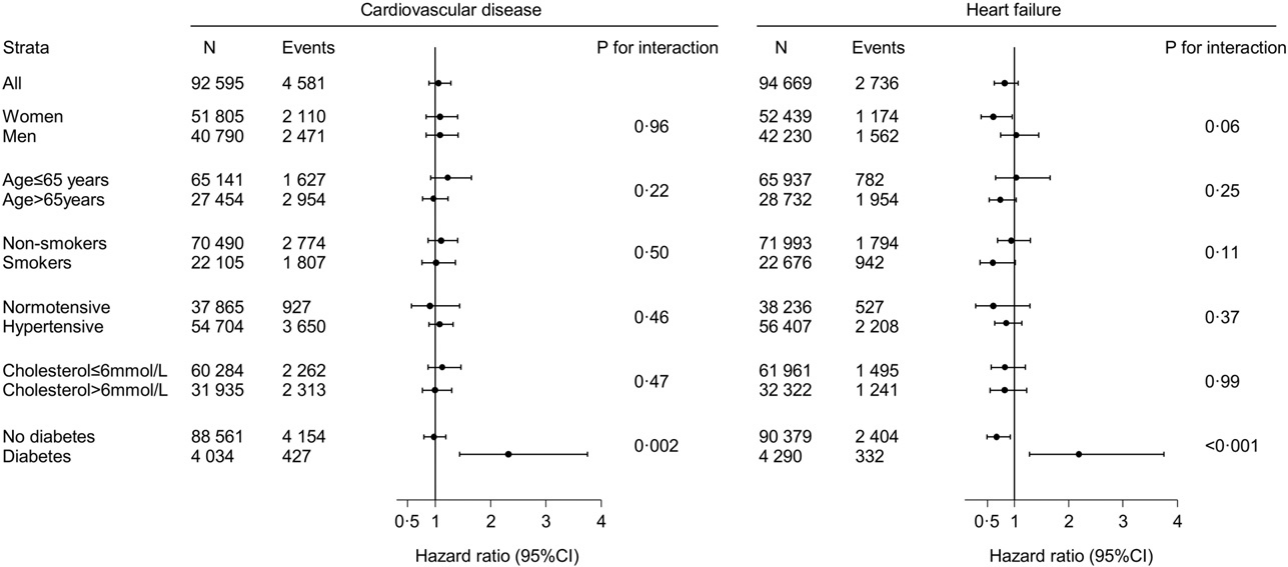
Association between SOD3 R213G genotype and cardiovascular disease and heart failure, stratified by risk factors. R213G genotype was heterozygotes (213RG) versus non-carriers (213RR). Cardiovascular disease was the composite endpoint of cardiovascular death, myocardial infarction, and ischemic stroke. Diabetes was baseline diabetes: self-reported diabetes, hospital diagnosis of diabetes prior to examination, non-fasting plasma glucose > 11 mmol/L at examination, or use of antidiabetic medication. Smokers were current smokers. All estimates were adjusted for age and study. Cholesterol was plasma total cholesterol. CI: confidence interval.

In order to assess if R213G genotype interacted with other known cardiovascular risk factors besides diabetes, we stratified analyses by gender, age, smoking status, hypertension and cholesterol levels, but no interactions except with diabetes were observed (Fig. 3). We found a total of 14 R213G homozygotes (0.01%) that we did not include in our analyses due to insufficient statistical power for homozygotes alone. However, in sensitivity analysis with R213G heterozygotes and homozygotes pooled results were similar as when examining heterozygotes alone (compare Supplemental Figs. 1 and 2). Additionally, when individuals with type 1 diabetes (n = 393) at baseline were excluded, results were similar (compare Supplemental Figs. 1 and 3). We could not perform the analyses for type 1 diabetes separately, due to too few R213G heterozygotes in this group.

## 4 Discussion

In this study of 95,857 white individuals from the Danish general population, including 4498 with diabetes, risk of cardiovascular disease and heart failure was higher in R213G heterozygotes versus non-carriers in diabetic subjects, but not in non-diabetic subjects.

Hyperglycemia, oxidative stress, and cardiovascular disease could be linked through different mechanisms. First, hyperglycemia may lead to increased superoxide radical formation in vascular endothelial cells and smooth muscle cells by NADPH oxidase through increased formation of mitochondrial reactive oxygen species (ROS) (Paneni et al., 2013) and advanced glycated end products (AGE) (Ago, T, et al., 2011, Goldin, A, et al., 2006). Second, superoxide radicals and other ROS can react with nitric oxide (NO), an important endothelial-derived vasodilator, decreasing NO bioavailability and generating the powerful oxidant peroxynitrite. This may lead to endothelial cell dysfunction or damage, which may promote both atherosclerosis and heart failure (Davignon, J and Ganz, P, 2004, Joshi, M, et al., 2014, Varga, ZV, et al., 2015). Third, higher levels of ROS can increase oxidative modifications of LDL, which increase LDL uptake by macrophages and lead to foam cell formation, a constituent of the atherosclerotic plaque (Maiolino et al., 2013). Additionally, oxidized LDL is thought to influence processes promoting the atherosclerotic process including activation of macrophages, smooth muscle cells, and platelet adhesion and aggregation (Maiolino et al., 2013). In diabetic subjects, heart failure often develops independently of other cardiovascular risk factors, suggesting a different pathogenesis of heart failure in diabetic than in non-diabetic individuals (Joshi et al., 2014). One possible mechanism could be hyperglycemia-induced myocardial oxidative stress leading to contractile dysfunction and microvascular changes with myocardial remodeling and fibrosis (Waddingham, MT, et al., 2015, Orasanu, G and Plutzky, J, 2009). Taken together, it seems biologically plausible that the SOD3 R213G variant, by drastically reducing tissue affinity of the SOD3 enzyme and leaving the vascular tissue with low protection against superoxide radicals, may increase the risk of cardiovascular disease and heart failure. Assuming this, the risk of cardiovascular disease and heart failure would be even higher among R213G homozygotes with diabetes, however, it is a rare genotype and we only identified 14 homozygotes, none of which had diabetes. Thus, we were not able to assess the effect of R213G homozygosity on cardiovascular disease and heart failure in diabetic subjects.

Previously, Mohammedi et al. found an association between the promotor variant rs2284659 in the *SOD3* gene, associated with higher plasma and presumably higher tissue levels of SOD3, and lower cardiovascular morbidity and mortality with a hazard ratio of 0·75 (95% CI: 0.59–0.94) for myocardial infarction and of 0.83 (0.69–0.99) for cardiovascular mortality in three prospective cohorts including 3921 type 1 and type 2 diabetic patients (Mohammedi et al., 2015). These findings indirectly support our findings as carriers of this promotor variant should have higher protection against superoxide radicals and decreased risk of cardiovascular disease and mortality. Also supporting our findings, Yamada et al. examined the association between the SOD3 R213G variant and mortality in 456 hemodialysis patients, of which 87 had diabetes, and found a higher mortality in R213G heterozygote diabetic subjects than in non-carriers (Yamada et al., 2000). In contrast, Ukkola et al. found no difference in prevalence of micro or macroangiopathy in 8 R213G heterozygotes among 222 type 2 diabetic subjects (Ukkola et al., 2001). However, with only a small number of R213G heterozygotes, the power to detect a modest effect may have been limited. Taken together, there seems to be emerging clinical evidence that SOD3 R213G heterozygosity may affect prognosis specifically among diabetic subjects. So far, only few cardiovascular risk factors selective to diabetes have been described. Interestingly, a genetic variation in the region of the *GLUL* gene on chromosome 1q25, rs10911021, has been robustly associated with coronary heart disease among diabetic subjects in a 3-stage GWAS in 4188 type 2 diabetic patients. Furthermore, this genetic variant was shown to affect the glutamate and glutamine metabolism in endothelial cells, and it is possible that the increased coronary heart disease risk in diabetic subjects could, at least in part, be the result of a limited availability of the antioxidant glutathione in these carriers (Qi et al., 2013).

A main strength of our study is the relative large number of R213G heterozygotes with and without diabetes. Also, we were able to examine the association between R213G heterozygotes and cardiovascular disease and heart failure in two large independent studies and found similar results. However, potential limitations to our study should be considered. First, we used cause of death in our composite endpoint of cardiovascular disease and it is possible that death from coronary heart disease is overestimated among diabetic subjects, due to physicians' preexisting knowledge on the association between diabetes and cardiovascular disease; importantly, however, this cannot explain the findings in the present study as genotyping and ascertainment of cardiovascular disease were done blinded to each other. Second, we pooled type 1 and type 2 diabetes in order to maximize statistical power; yet, results were similar after excluding type 1 diabetes. Furthermore, the hypothesis that increased oxidative stress from hyperglycemia leads to cardiovascular disease, and thus, that the two types of diabetes share a common pathway to cardiovascular disease supports pooling type 1 and type 2 diabetes. Lastly, since all individuals were whites of Danish descent, our results may not necessarily apply to other ethnicities; nevertheless, this also minimizes the risk of population stratification and we are not aware of findings that suggest our results should not apply to other ethnicities.

In conclusion, risk of cardiovascular disease and heart failure was higher in R213G heterozygotes versus non-carriers in diabetic subjects, but not in non-diabetic subjects. These findings may have both biological and clinical implications. First, this could provide further insight on the pathophysiology of cardiovascular disease and heart failure in diabetes. Second, if our findings are replicated in future studies, *SOD3* R213G heterozygosity could be used as a prognostic marker.

## Declaration of Interests

The authors declare to have no conflicts of interest.

## Contributions

CJK, SA and BGN designed the study, CJK analyzed the data, CJK, SA and BGN interpreted the data and wrote the manuscript. All of the authors read and approved the final manuscript.

## Figures and Tables

**Table 1:** Baseline characteristics by SOD3 R213G (rs1799895) genotype stratified by baseline diabetes status in the Copenhagen City Heart Study and the Copenhagen General Population Study combined.

	No baseline diabetes			Baseline diabetes		
Non-carriers (213RR)	Heterozygotes (213RG)	p	Non-carriers (213RR)	Heterozygotes (213RG)	p
N = 89,145	N = 2214		N = 4376	N = 122	
Men, (%)	39,463 (44)	980 (44)	0.93	2456 (56)	71 (58)	0.65
Age, years	57 (47–67)	57 (47–66)	0.32	66 (58–73)	65 (60–72)	0.99
Ever smoker	54,709 (61)	1336 (60)	0.32	2959 (68)	74 (61)	0.11
Height, m	1.7 (1.6–1.8)	1.7 (1.6–1.8)	0.96	1.7 (1.6–1.8)	1.7 (1.6–1.8)	0.90
Cumulative tobacco consumption^a^, pack-years	16 (6–30)	16 (6–30)	0.25	25 (11–40)	28 (15–50)	0.08
BMI, kg/m^2^	25 (23–28)	25 (23–28)	0.55	28 (25–32)	29 (26–33)	0.15
Alcohol consumption, units/week	8 (4–15)	8 (3–15)	0.15	8 (3–15)	8 (3–14)	0.84
High income	34,138 (38)	886 (40)	0.10	900 (21)	28 (23)	0.52
High physical activity (leisure time)	43,586 (49)	1078 (49)	0.85	1690 (39)	43 (35)	0.45
High physical activity (at work)	17,496 (20)	436 (20)	0.94	694 (16)	22 (18)	0.52
Systolic blood pressure, mm Hg	139 (125–154)	139 (125–154)	0.68	146 (132–160)	150 (139–164)	0.04^d^
LDL cholesterol, mmol/L	3.3 (2.6–3.9)	3.3 (2.6–4.0)	0.15	2.6 (1.9–3.3)	2.5 (1.9–3.3)	0.69
HDL cholesterol, mmol/L	1.6 (1.3–1.9)	1.6 (1.2–1.9)	0.86	1.4 (1.1–1.7)	1.4 (1.1–1.6)	0.43
Triglycerides, mmol/L	1.4 (0.97–2.1)	1.4 (0.95–2.1)	0.21	1.8 (1.2–2.7)	1.9 (1.2–2.7)	0.71
CRP, mg/L	1.4 (1.0–2.4)	1.4 (1.0–2.4)	0.34	1.9 (1.2–3.7)	1.9 (1.2–3.5)	0.70
Cholesterol-lowering medication	7773 (9)	197 (9)	0.77	2053 (47)	57 (47)	0.97
Glucose, mmol/L	5.1 (4.7–5.6)	5.1 (4.7–5.6)	0.44	6.7 (5.5–9.6)	6.9 (5.5–9.6)	0.97
Insulin	0	0	1.0	801 (18)	25 (20)	0.54
Oral antidiabetic medications	0	0	1.0	2030 (46)	68 (56)	0.04^d^
Antihypertensive medication	15,505 (17)	375 (17)	0.58	2385 (55)	67 (55)	0.93
Cardiac medications	3792 (4)	88 (4)	0.52	635 (15)	14 (12)	0.35
Anticoagulant therapy^b^	8691 (11)	219 (11)	0.87	1628 (41)	47 (42)	0.76
SOD3^c^, ng/mL (SD)	142 (46)⁎	1285 (395)⁎⁎	< 0.001	132 (36)⁎⁎⁎	1110 (318)⁎⁎⁎⁎	< 0.001

Continuous variables are shown as median (interquartile range). Categorical data are n (%). P is from Kruskal-Wallis test. Baseline diabetes: self-reported diabetes of any type, hospital diagnosis of diabetes prior to examination, non-fasting plasma glucose > 11 mmol/L at examination, or use of antidiabetic medication. BMI: body mass index. LDL: low density lipoprotein. HDL: high density lipoprotein. CRP: C-reactive protein. SOD3: superoxide dismutase 3.

a Among ever smokers only.

b Only available in the CGPS.

c Plasma SOD3 measured in the Copenhagen City Heart Study only, shown are mean (SD).

d Not significant after Bonferroni correction for multiple testing.

⁎ N = 2057 individuals.

⁎⁎ N = 190 individuals.

⁎⁎⁎ N = 135 individuals.

⁎⁎⁎⁎ N = 11 individuals.
