# Supplementary material for: Genetically Low Antioxidant Protection and Risk of Cardiovascular Disease and Heart Failure in Diabetic Subjects
Source: eBioMedicine. 2015 Nov 14;2(12):2010–5. doi: 10.1016/j.ebiom.2015.11.026 (PMC4703764; doi:10.1016/j.ebiom.2015.11.026)
Supplement: Supplementary file 1 — Supplementary figures. [file mmc1.pdf]

Cardiovascular disease

Combined studies	N	Events	Hazard ratio (95%CI)	P for interaction
All participants				
Non-carriers	90 339	4 465	1	
Heterozygotes	2 256	116	1·05(0·88-1·57)	
No diabetes at baseline				
Non-carriers	86 414	4 056	1	
Heterozygotes	2 147	98	0·97(0·80-1·19)	0·002
Diabetes at baseline				
Non-carriers	3 925	409	1	
Heterozygotes	109	18	2·32(1·44-3·75)	
**Copenhagen City Heart Study**				
All participants				
Non-carriers	8 250	2 019	1	
Heterozygotes	199	57	1·11(0·85-1·45)	
No diabetes at baseline				
Non-carriers	7 901	1 850	1	
Heterozygotes	188	49	1·02(0·76-1·35)	0·003
Diabetes at baseline				
Non-carriers	349	169	1	
Heterozygotes	11	8	3·40(1·64-7·05)	
**Copenhagen General Population Study**				
All participants				
Non-carriers	82 089	2 446	1	
Heterozygotes	2 057	59	1·01(0·78-1·31)	
No diabetes at baseline				
Non-carriers	78 513	2 206	1	
Heterozygotes	1 959	49	0·92(0·69-1·23)	0·06
Diabetes at baseline				
Non-carriers	3 576	240	1	
Heterozygotes	98	10	1·79(0·94-3·42)	

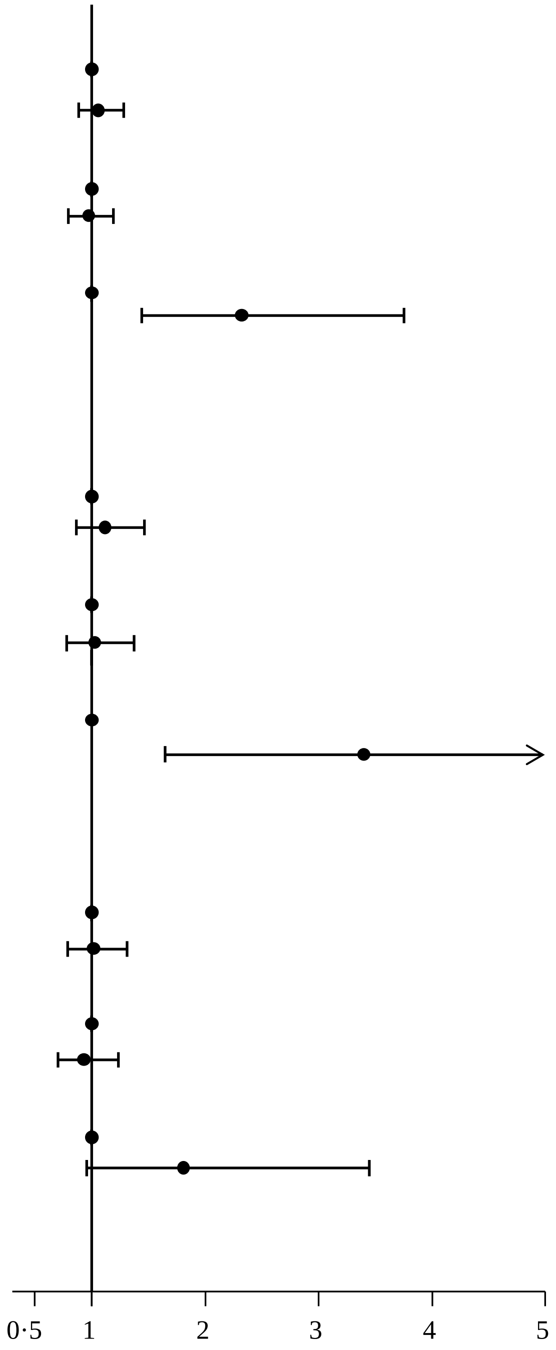

Heart failure

Combined studies	N	Events	Hazard ratio (95% CI)	P for interaction
All participants				
Non-carriers	92 364	2 682	1	
Heterozygotes	2 305	54	0·82(0·62-1·07)	
No diabetes at baseline				
Non-carriers	88 192	2 364	1	
Heterozygotes	2 187	40	0·68(0·49-0·92)	<0·001
Diabetes at baseline				
Non-carriers	4 172	318	1	
Heterozygotes	118	14	2·19(1·28-3·76)	
**Copenhagen City Heart Study**				
All participants				
Non-carriers	8 559	1 226	1	
Heterozygotes	204	26	0·86(0·58-1·27)	
No diabetes at baseline				
Non-carriers	8 179	1 110	1	
Heterozygotes	194	22	0·78(0·51-1·19)	
Diabetes at baseline				0.02
Non-carriers	380	116	1	
Heterozygotes	10	4	2·86(1·03-7·89)	
**Copenhagen General Population Study**				
All participants				
Non-carriers	83 805	1 456	1	
Heterozygotes	2 101	28	0·81(0·56-1·17)	
No diabetes at baseline				
Non-carriers	80 013	1 254	1	
Heterozygotes	1 993	18	0·59(0·37-0·94)	
Diabetes at baseline				0·004
Non-carriers	3 792	202	1	
Heterozygotes	108	10	2·06(1·09-3·91)	

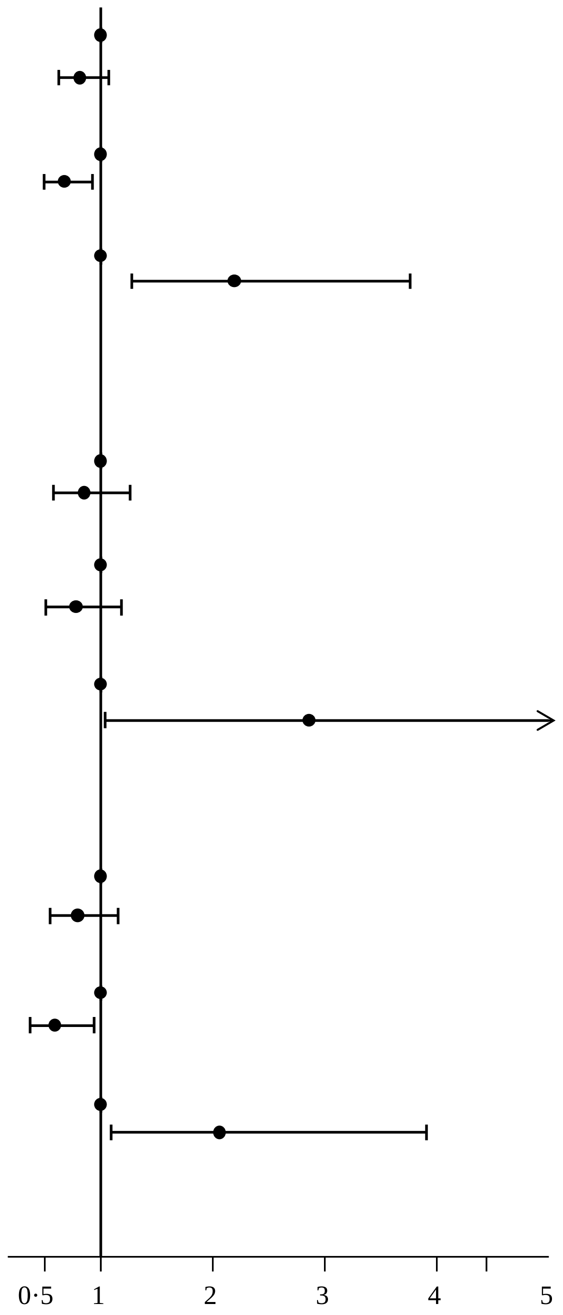

Supplemental Figure 1. SOD3 R213G genotype and risk of cardiovascular disease and heart failure in the combined studies and in the two studies separately, stratified by diabetes status. 3 262 individuals had experienced a cardiovascular event and 1188 individuals had experienced heart failure prior to examination and were not included in the analyses. Baseline diabetes: self-reported diabetes, hospital diagnosis of diabetes prior to examination, non-fasting plasma glucose >11mmol/L at examination, and/or use of antidiabetic medication. All estimates were adjusted for age; the combined studies were also adjusted for study. Cardiovascular disease was the composite endpoint of cardiovascular death, myocardial infarction, and ischemic stroke. CI: confidence interval.
