# Supplementary material for: Genetically Low Antioxidant Protection and Risk of Cardiovascular Disease and Heart Failure in Diabetic Subjects
Source: eBioMedicine. 2015 Nov 14;2(12):2010–5. doi: 10.1016/j.ebiom.2015.11.026 (PMC4703764; doi:10.1016/j.ebiom.2015.11.026)
Supplement: Supplementary file 2 — Supplementary figures. [file mmc2.pdf]

Cardiovascular disease

Combined studies	N	Events	Hazard ratio (95% CI)	P for interaction
All participants				
Non-carriers	90 339	4 465	1	
Hetero+homo	2 267	116	1·05(0·87-1·27)	
No diabetes at baseline				
Non-carriers	86 414	4 056	1	
Hetero+homo	2 158	98	0·96(0·79-1·18)	0·001
Diabetes at baseline				
Non-carriers	3 925	409	1	
Hetero+homo	109	18	2·32(1·44-3·75)	
**Copenhagen City Heart Study**				
All participants				
Non-carriers	8 250	2 019	1	
Hetero+homo	200	57	1·11(0·85-1·44)	
No diabetes at baseline				
Non-carriers	7 910	1 850	1	
Hetero+homo	189	49	1·02(0·77-1·35)	0·002
Diabetes at baseline				
Non-carriers	349	169	1	
Hetero+homo	11	8	3·41(1·64-7·05)	
**Copenhagen General Population Study**				
All participants				
Non-carriers	82 089	2 446	1	
Hetero+homo	2 067	59	1·01(0·78-1·30)	
No diabetes at baseline				
Non-carriers	78 513	2 206	1	
Hetero+homo	1 969	49	0·92(0·70-1·22)	0·06
Diabetes at baseline				
Non-carriers	3 576	240	1	
Hetero+homo	98	10	1·81(0·96-3·45)	

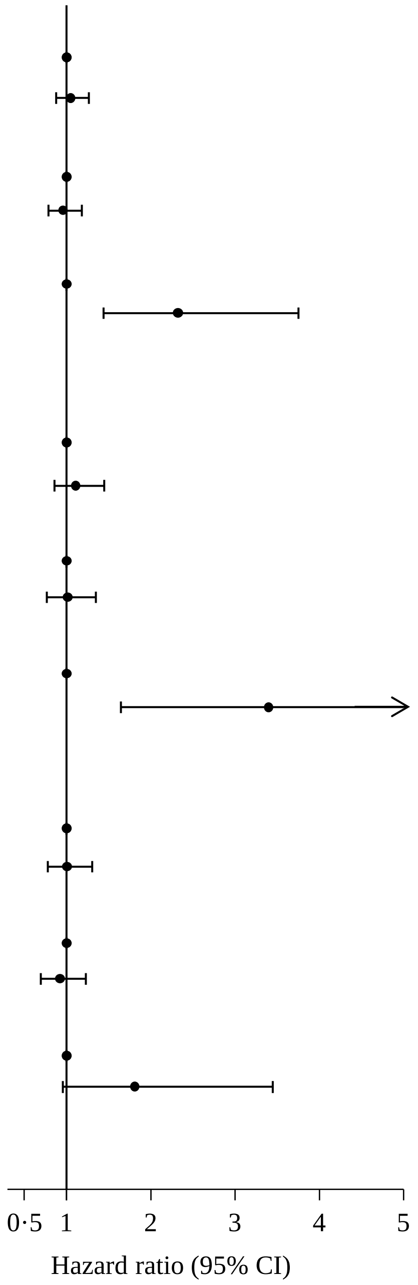

Heart failure

Combined studies	N	Events	Hazard ratio (95% CI)	P for interaction
All participants				
Non-carriers	92 364	2 682	1	
Hetero+homo	2 318	55	0·82(0·63-1·08)	
No diabetes at baseline				
Non-carriers	88 192	2 364	1	
Hetero+homo	2 220	41	0·68(0·50-0·93)	
Diabetes				<0·001
Non-carriers	4 172	318	1	
Hetero+homo	118	14	2·19(1·28-3·76)	
**Copenhagen City Heart Study**				
All participants				
Non-carriers	8 559	1 226	1	
Hetero+homo	206	26	0·84(0·57-1·24)	
No diabetes at baseline				
Non-carriers	8 179	1 110	1	
Hetero+homo	196	22	0·76(0·50-1·16)	
Diabetes at baseline				0·02
Non-carriers	380	116	1	
Hetero+homo	10	4	2·86(1·03-7·89)	
**Copenhagen General Population Study**				
All participants				
Non-carriers	83 805	1 456	1	
Hetero+homo	2 112	29	0·82(0·57-1·18)	
No diabetes at baseline				
Non-carriers	80 013	1 254	1	
Hetero+homo	2 004	19	0·62(0·39-0·98)	
Diabetes at baseline				0·005
Non-carriers	3 792	202	1	
Hetero+homo	108	10	2·06(1·09-3·90)	

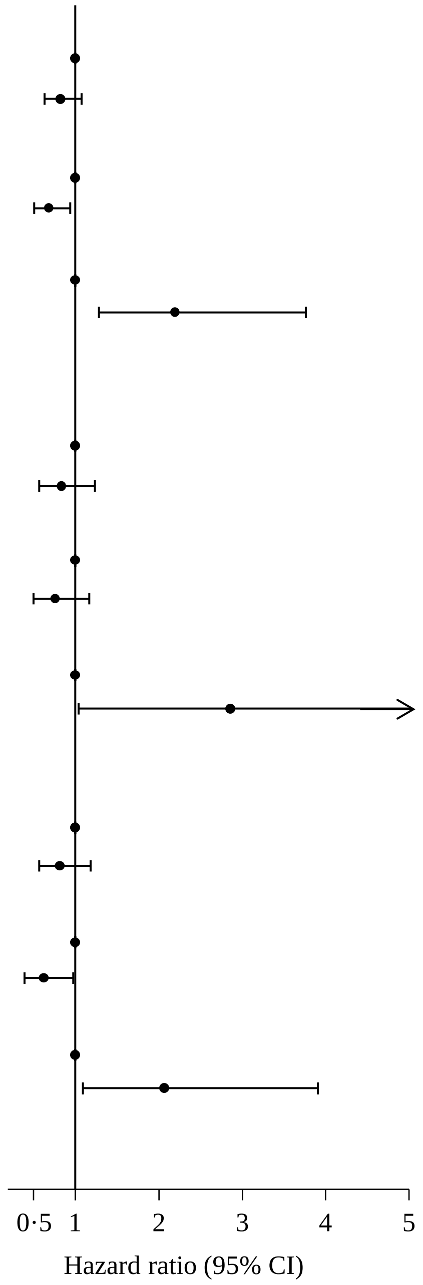

Supplemental Figure 2. SOD3 R213G genotype and risk of cardiovascular disease and heart failure, stratified by baseline diabetes status. Hetero+homo were R213G heterozygotes and homozygotes combined. Baseline diabetes: self-reported diabetes, hospital diagnosis of diabetes prior to examination, non-fasting plasma glucose >11mmol/L at examination, and/or use of antidiabetic medication. All estimates were adjusted for age; the combined studies were also adjusted for study. Cardiovascular disease was the composite endpoint of cardiovascular death, myocardial infarction, and ischemic stroke. CI: confidence interval.
