# Supplementary material for: Genetically Low Antioxidant Protection and Risk of Cardiovascular Disease and Heart Failure in Diabetic Subjects
Source: eBioMedicine. 2015 Nov 14;2(12):2010–5. doi: 10.1016/j.ebiom.2015.11.026 (PMC4703764; doi:10.1016/j.ebiom.2015.11.026)
Supplement: Supplementary file 3 — Supplementary figures. [file mmc3.pptx]

## Slide 1
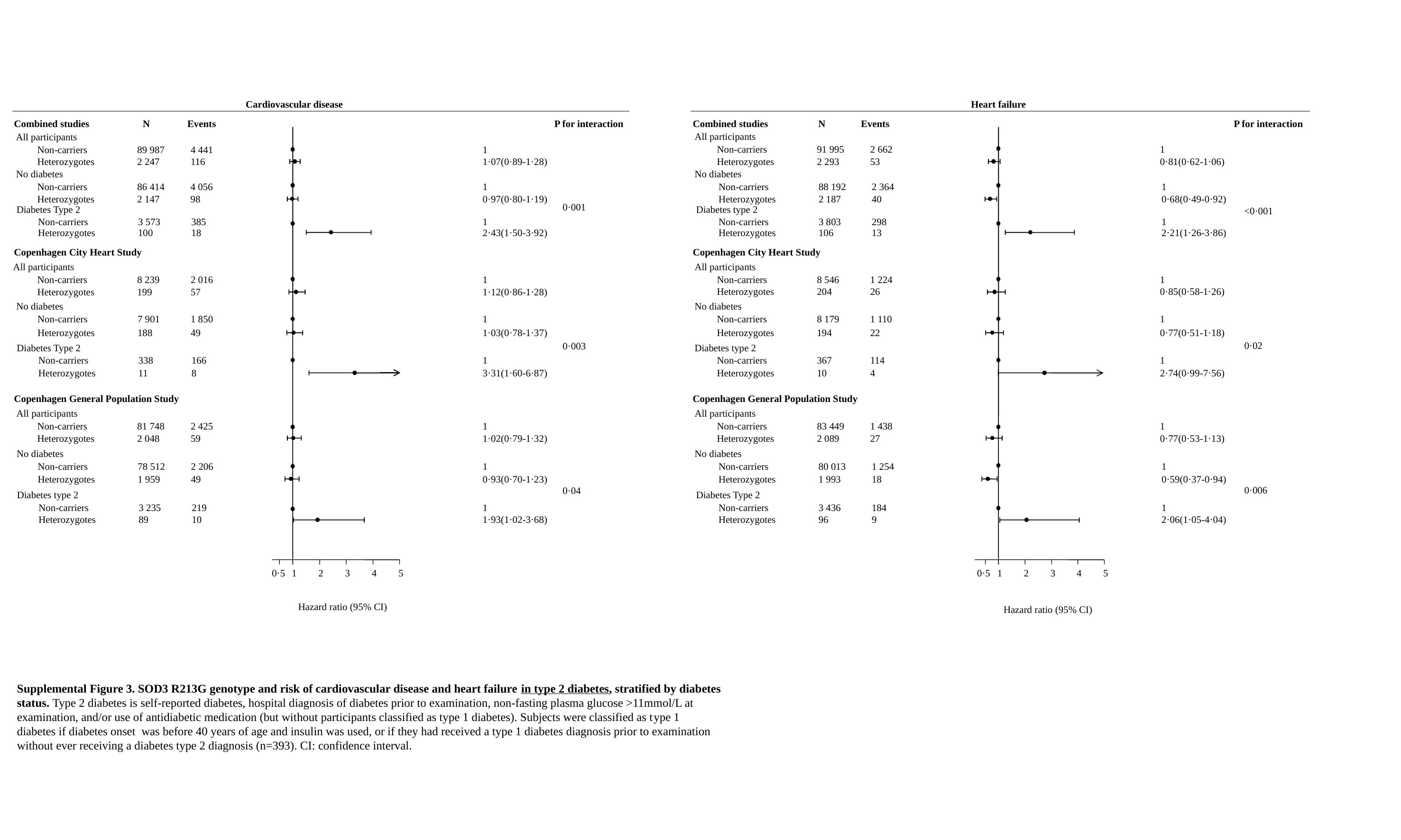

Cardiovascular disease
Heart failure
Combined studies
N
Events
P for interaction
Combined studies
N
Events
P for interaction
All participants
All participants
Non-carriers
91 995
2 662
1
Non-carriers
89 987
4 441
1
Heterozygotes
2 293
53
0·81(0·62-1·06)
Heterozygotes
2 247
116
1·07(0·89-1·28)
No diabetes
No diabetes
Non-carriers
86 414
4 056
1
Non-carriers
88 192
2 364
1
Heterozygotes
2 147
98
0·97(0·80-1·19)
Heterozygotes
2 187
40
0·68(0·49-0·92)
0·001
Diabetes Type 2
Diabetes type 2
<0·001
Non-carriers
3 573
385
1
Non-carriers
3 803
298
1
Heterozygotes
100
18
2·43(1·50-3·92)
Heterozygotes
106
13
2·21(1·26-3·86)
Copenhagen City Heart Study
Copenhagen City Heart Study
All participants
All participants
Non-carriers
8 546
1 224
1
Non-carriers
8 239
2 016
1
Heterozygotes
204
26
0·85(0·58-1·26)
Heterozygotes
199
57
1·12(0·86-1·28)
No diabetes
No diabetes
Non-carriers
7 901
1 850
1
Non-carriers
8 179
1 110
1
Heterozygotes
188
49
1·03(0·78-1·37)
Heterozygotes
194
22
0·77(0·51-1·18)
0·02
0·003
Diabetes Type 2
Diabetes type 2
Non-carriers
338
166
1
Non-carriers
367
114
1
Heterozygotes
11
8
3·31(1·60-6·87)
Heterozygotes
10
4
2·74(0·99-7·56)
Copenhagen General Population Study
Copenhagen General Population Study
All participants
All participants
Non-carriers
83 449
1 438
1
Non-carriers
81 748
2 425
1
Heterozygotes
2 089
27
0·77(0·53-1·13)
Heterozygotes
2 048
59
1·02(0·79-1·32)
No diabetes
No diabetes
Non-carriers
78 512
2 206
1
Non-carriers
80 013
1 254
1
Heterozygotes
1 959
49
0·93(0·70-1·23)
Heterozygotes
1 993
18
0·59(0·37-0·94)
0·006
0·04
Diabetes type 2
Diabetes Type 2
Non-carriers
3 235
219
1
Non-carriers
3 436
184
1
Heterozygotes
89
10
1·93(1·02-3·68)
Heterozygotes
96
9
2·06(1·05-4·04)
0·5
1
2
3
4
5
0·5
1
2
3
4
5
Hazard ratio (95% CI)
Hazard ratio (95% CI)
Supplemental Figure 3. SOD3 R213G genotype and risk of cardiovascular disease and heart failure in type 2 diabetes, stratified by diabetes status. Type 2 diabetes is self-reported diabetes, hospital diagnosis of diabetes prior to examination, non-fasting plasma glucose >11mmol/L at examination, and/or use of antidiabetic medication (but without participants classified as type 1 diabetes). Subjects were classified as type 1 diabetes if diabetes onset was before 40 years of age and insulin was used, or if they had received a type 1 diabetes diagnosis prior to examination without ever receiving a diabetes type 2 diagnosis (n=393). CI: confidence interval.
